# Health of the Officers Employed in Her Majesty's Post Office during the Year 1857

**Published:** 1858-04

**Authors:** Waller Lewis

**Affiliations:** Medical Officer to H.M. Post Office.


### ORIGINAL COMMUNICATIONS.

### HEALTH OF THE OFFICERS EMPLOYED IN HER MAJESTY'S POST OFFICE DURING THE YEAR 1857.

By WALLER LEWIS, M.B.Cantab., Medical Officer to H.M. Post Office.

Medical Department, General Post Office, Feb. 27, 1858.

SIR,—I beg to present my third Annual Report on the Health of the Officers of this Department.

NUMBER OF OFFICERS UNDER MEDICAL CHARGE.—As there have been no epidemics among the officers during the past year, the heads of departments and clerks with salaries exceeding £150 per annum, comprised in class A, have not come under my care. Their number was 256.

In class B, comprising clerks and inspectors with salaries not exceeding £150 per annum, there were 293.

In class C, which includes all the other officers in the minor establishment attached to the chief, and Money-order Offices, there were 1375, making in the aggregate 1668 officers under my medical charge.

But so many changes take place in the course of the year, more particularly among the officers of the minor establishment, that as many as 1763 persons have had the privilege of applying for assistance to the medical officer during the year. As a considerable proportion of the officers constituting the difference between the permanent staff of 1668 and the total number of officers who have been temporarily attached, always help to increase the amount of cases of illness, the number may be fairly averaged as 1700 for the entire year.

CANDIDATES EXAMINED.—*Clerks.* During the year, 43 candidates for clerkships presented themselves for medical examination, and of these, three were rejected as unfit.

*Minor Officials.* The number of candidates for the office of letter-carrier during the year was 319. They were thus disposed of:—

| | | |
|---|---|---|
| Appointed | - - - - - - - | 168 |
| Found disqualified on examination by | Civil Service Commissioners | 89 |
| „ „ | Medical Officer - - | 36 |
| „ „ | being above age - - | 10 |
| „ „ | being under age - - | 5 |
| Declined the appointment | - - - - | 7 |
| Characters unsatisfactory | - - - - | 2 |
| Nominations cancelled | - - - - - | 2 |
| | | 319 |

This shows that, while 11 per cent. of the candidates were rejected on grounds of physical incapacity, 89 of the 257 who presented themselves before the Civil Service Commissioners, or 34 per cent., were disqualified for want of the requisite amount of education.

*Previous occupations of candidates for Letter-carrierships.* Observing that a large number of the candidates who present themselves for medical examination, are small, slightly made, and sickly looking, I have obtained information of the previous employments of the last 329.

They were as follows :—

| | |
|---|---|
| Domestic and other servants | 60 |
| Porters and messengers | 38 |
| Clerks in counting-houses, etc. | 20 |
| Schoolmasters | 2 |
| Farmers and labourers | 10 |
| Soldiers and sailors | 14 |
| Workers in metals | 11 |
| Grocers | 13 |
| Tailors | 14 |
| Drapers and shopmen | 13 |
| Painters and plasterers | 9 |
| Shoemakers | 7 |
| Butchers | 9 |
| Bakers | 7 |
| Gardeners | 9 |
| Policemen | 5 |
| Booksellers, printers, etc. | 6 |
| Weavers | 3 |
| Carpenters and packers, etc. | 6 |
| Druggists | 3 |
| Tobacconists | 2 |
| Fishmonger | 1 |
| Publican | 1 |
| Coiner at Mint | 1 |
| Doll maker | 1 |
| Pipe maker | 1 |
| Milkman | 1 |
| Sand dealer | 1 |
| Moulders, furriers, and leather cutters | 7 |
| Unknown | 54 |
| | 329 |

It will be seen from this list how small a proportion of the letter-carriers, sorters, and stampers, employed in the department, is derived from agricultural labourers, or from persons who have been accustomed to healthy out-of-door employment. The large proportion of domestic servants, clerks in counting-houses, tailors, drapers' and grocers' assistants, and other shopmen, accounts for the generally debilitated and under-sized condition of the present class of candidates. I am informed by officers who have known the department for many years, that formerly a very different class of persons presented themselves for employment as postmen. Many agricultural labourers, and others of strong muscular development constituted the general run of applicants, whereas, for some few years past, these are the rare exceptions.

The consequence of this state of things is, that there is more illness among the officers than the nature of their work might be expected to give rise to. That the occupation of the men employed in the minor establishment is by no means necessarily injurious to health I have shown in a special report on the domiciliary condition of the men. Instances are there given of officers who have been actively employed in the service for periods varying from thirty-five to forty-six years. During the last ten years seven men have left the service, all of whom had been employed as letter-carriers for more than half a century: and in the course of the last five years nineteen men have quitted the department, who had served between forty and fifty years. But though the work is not detrimental to men of average constitutional strength, and of fair muscular development, and with sound organs of breathing and circulation, it is quite the contrary with persons of impaired health, or who are undersized in stature or weakly framed. I have therefore proposed that his Grace the Postmaster-General should sanction the medical officer rejecting as unfit for the work, not only such candidates as are actually labouring under disease, but also such as are not found by certain specified tests equal to a stated amount of corporeal exertion. The injury done to candidates themselves, by admitting those that have delicate lungs, is shown by the subjoined tables of mortality. It will be seen that out of twenty deaths in the department, including clerks, ten, or exactly half, have been victims of consumption.

That this is a very large proportion of deaths from this disease is proved by the fact, that while the total number of deaths in the city of London during the year 1857 was 2942, but 356 of that number, or less than one-eighth part, were caused by phthisis. Very nearly the same proportion obtains for the whole metropolis, where, on an average of seventeen years, the deaths from decline were 13·173 to every 100 deaths. Of the deaths from all causes of persons aged twenty years (which is about the average age at which persons enter as officers of this department) and upwards in London, the proportion of those dying from phthisis is 18·1 per cent. on an average of the three years 1853-55.

In all of the ten fatal cases, whose history during life I was enabled to investigate, the disease proved to be hereditary, showing, that if proper care had been taken on the application of those officers for admittance while candidates, the greater number, if not all of them, would have been rejected. All of these officers had been in the department for some years previous to the appointment of an examining medical officer. I am sorry to state that there are still on my list numerous patients, (a very large proportion of whom are Irish), suffering from this fearful scourge, all of whose days are numbered, and who will, for some time to come, help to swell inordinately the annual list of deaths. In my opinion, the utmost strictness should be used in excluding, not only such candidates as are actually, at the time of presenting themselves, suffering from this complaint, but also such as show a strongly marked tendency thereto, especially when this is combined with a weak frame of body. The average age of the consumptive patients, at the time of their death, was 29.

General Health. The diminution in the number of cases of illness and in their duration, proves that the general health of the whole establishment has been good. Compared with the year 1856, the past year exhibits a favourable contrast, notwithstanding an increase in the general mortality. In the year 1856, among 1500 officers there had occurred 1771 cases of illness, or 118 per cent., while during the past year, among 1700 officers, being an increase of 200, there have been but 1403 cases, or 82 per cent. This shows a diminution of 36 per cent. of sickness in 1857 compared with 1856.

The following table shows the number of clerks and other officers who presented themselves for official medical assistance during the year 1857, specifying some of the more common complaints :—

TABLE I.

*Sickness during the Year* 1857.

| Months. | Clerks | Others. | Total. | Diarr. | Rheumatism. | Boils. | Sore throat. |
|---|---|---|---|---|---|---|---|
| January . . . . . | 10 | 110 | 120 | 3 | 10 | 3 | 5 |
| February . . . . | 15 | 118 | 133 | 6 | 7 | 5 | 1 |
| March . . . . . | 6 | 96 | 102 | 4 | 9 | 4 | 7 |
| April . . . . . . | 4 | 84 | 88 | 3 | 5 | 2 | 5 |
| May . . . . . . . . | 13 | 87 | 100 | 6 | 8 | 3 | 9 |
| June . . . . . . | 15 | 107 | 122 | 20 | 5 | 5 | 3 |
| July . . . . . . . . | 21 | 133 | 154 | 63 | 7 | 5 | 7 |
| August . . . . . | 17 | 144 | 161 | 66 | 9 | 5 | 2 |
| September . . . | 16 | 106 | 122 | 21 | 9 | .. | 5 |
| October . . . . . | 8 | 106 | 114 | 13 | 11 | 3 | 1 |
| November . . . | 19 | 85 | 104 | 6 | 3 | 2 | 5 |
| December . . . | 7 | 76 | 83 | 4 | 9 | 1 | 5 |
| Total . . . . | 151 | 1252 | 1403 | 215 | 92 | 38 | 55 |

524 visits were paid to officers at their own houses, when too ill to attend at the office. I also visited officers at York, Derby, Rugby, and Newcastle, who had met with injuries in the execution of their duties.

Epidemics. There have been no special epidemics during the year. Sixty-two branch and district offices have been supplied with medicine for the immediate and efficient treatment of the usual summer and autumnal diarrhœa, so frequent among the letter-carriers. By means of careful directions to the charge-takers and inspectors at the various offices, great numbers of the men have been relieved at once, and in most instances they have been enabled to continue their duties uninterruptedly. Upwards of 250 gallons of the medicine have been thus dispensed.

Average duration of Illness.—*Clerks.* One hundred and fifty-one clerks presented themselves at the office for treatment during the year. This number includes a few in class A, who were labouring under diarrhœa. The total number of heads of departments, clerks, inspectors, etc., comprehended under classes A and B, who have been off duty from illness, is 465, and the total amount of such absences is 4,919 days, making an average of ten and a half days each.

If nineteen of these cases, however, (some of which were absences not really caused by illness, seven ending in retirement from the department, and the rest being followed by dismissal), which have each exceeded three months in duration, and have engrossed between them 2,328 of the whole number of days, be deducted from the gross amount, the mean average duration of each case of illness among those clerks who were absent, will be about five days and three quarters.

*Minor Establishment.* The total amount of absence from duty in days, on account of illness in class C, was 12,888 days. This will give a mean average duration of ten days for each officer who absented himself. But this amount of absence is unduly swollen by the prolonged absence of twenty-one officers who have been away 2875 days. As none of these will return to the department, and their places have been filled up by fresh appointments, their absence should be deducted from the gross amount. The average duration of each case will thus be reduced to about eight days. But a large proportion even of this reduced amount of absence is caused by few cases. Fifteen officers have been absent many months consecutively, one for more than eleven months.

*General body of Officers.* Of the 2019 officers who have been attached to the chief and Money-order offices during some portion of the year, 892 have been at some time absent from duty on account of illness. The average number of days that any officer was off duty from this cause, including in the calculation the whole body of officers, was about ten days. But if correction be made for the forty exceptional cases above alluded to, the average total absence will be six days and two-thirds. In other words, every officer in the department has, on the average, been absent from duty nearly a week on account of ill-health.

DEATHS. These are represented in the two tables subjoined.

TABLE II.

*Deaths during the Year* 1857. *Clerks, Inspectors, etc.*

| Disease. | Age at Death. | | | |
|---|---|---|---|---|
| | 30 to 40. | 40 to 50. | 50 to 60. | Total. |
| Consumption .......... | 3 | .. | .. | 3 |
| Inflammation of bladder | .. | .. | 1 | 1 |
| Typhus .............. | 1 | .. | .. | 1 |
| Total .......... | 4 | .. | 1 | 5 |

As these five deaths took place among a body numbering 549, it follows that the mortality among the heads of departments, clerks, inspectors, etc., was at the rate of 9 per 1000.

TABLE III.

*Deaths in Class C.*

(*Letter Carriers, Sorters, Messengers, Stampers, Porters, Labourers, Firemen, Constables, and Female Domestics.*)

| Disease. | Age at Death. | | | | | |
|---|---|---|---|---|---|---|
| | 20 to 30. | 30 to 40. | 40 to 50. | 50 to 60. | 60 to 70. | Total. |
| Consumption .......... | 1 | 1 | 5 | .. | .. | 7 |
| Inflammation of brain .. | 1 | .. | .. | .. | .. | 1 |
| Acute rheumatism ..... | 1 | .. | .. | .. | .. | 1 |
| Bronchitis & emphysema | 1 | .. | .. | .. | .. | 1 |
| Abscess of liver ....... | .. | .. | 1 | .. | .. | 1 |
| Disease of heart ...... | .. | .. | 1 | 1 | .. | 2 |
| Mortification of leg .... | .. | .. | .. | .. | 1 | 1 |
| Cancer of jaw ......... | .. | 1 | .. | .. | .. | 1 |
| Total .......... | 4 | 2 | 7 | 1 | 1 | 15 |

The number of officers among whom these fifteen deaths occurred being 1375, it follows that the mortality during the year was rather under 11 per 1000. The average mortality for the two years 1856 and 1857, taken together, amounted to

9 per 1000. I have compared this rate with the mortality in the same class (C) in each of the three years 1851, 1852, and 1853, omitting the year 1854, as it was a year during which epidemic cholera existed in London. From the statistics furnished to me, it appears, that those three years show the following mortality per 1000:—

| | |
|---|---|
| In 1851 - - - | 14·5 |
| 1852 - - - | 18· |
| 1853 - - - | 12·3 |

How far the very considerable diminution in the number of deaths in the two years just elapsed may be due to keeping a stricter surveillance over the admission of candidates than was practised before an official medical authority formed part of the staff of the department, and how far that improvement is due to other sanitary causes, it is impossible to state with any approach to accuracy.

Officers retired from the Service, and pensioned. During the year, forty-six officers have been superannuated; and thirty-two have been dismissed for bad conduct.

On the 31st of December, 1857, the number of officers receiving pensions for former services in this department was 225, exclusive of retired country postmasters, and officers who had been attached to the packet service. There had been fourteen deaths during the year among the superannuated officers ; a mortality of six per cent.

If a calculation of the total mortality, both among the efficient and non-efficient branches of the department, for the year, be now made, the numbers will stand thus:—

| | |
|---|---|
| Number of officers in Class A. - - | 256 |
| „ „ B. - - | 293 |
| „ „ C. - - | 1,700 |
| „ retired officers - - | 239 |
| | 2,488 |

among whom thirty-four deaths took place, a mortality of rather less than 14 per 1000.

These figures contrast favourably with the returns which the Registrar-General has transmitted to me for the three years 1853-5. From these, it appears that the annual average mortality in London of males aged twenty and upwards, is 24·2 per 1000.

The rate of deaths among the officers on active service being just 10 per 1000, while that among the superannuated officers was 60 per 1000, it follows, that the mortality was six times as great among the non-efficient, as among the men doing active duty.

*Age of Superannuated Officers on Retirement.* The officer on the present list of pensioners who retired earliest in life, was but twenty-eight years of age when he left the service; and the oldest did not leave till he had passed his seventy-seventh year. The average age of the officers at the time of their retirement was fifty-five.

*Length of Service.* One clerk, (the officer above alluded to as being but twenty-eight years of age on retirement), was allowed to leave on account of incurable disease, after only nine years' service; while several served fifty, and one more than fifty-one years, before applying for superannuation. The average length of service before retiring was nearly thirty years.

*Present age of Annuitants.* The ages of retired officers on the 31st December last, varied from twenty-eight to eighty-four. Their average age was fifty-nine years and three-quarters.

*Former status of retired Officers.* Of the 225 superannuated officers surviving on the 31st December, 1857, 53 had been heads of departments, clerks, inspectors, and other superior officers, while the remaining 172 were letter-carriers, messengers, sorters, and mail-guards.

*Causes of retirement.* The causes of retirement may be thus arranged:—

| | |
|---|---|
| Age, general debility, and broken-down constitution - - | 93 |
| Impaired vision - - - - - - | 24 |
| Diseases of brain and nervous system - - - | 18 |
| Chronic rheumatism - - - - - - | 14 |
| Diseases of the lungs - - - - - | 13 |
| „ heart - - - - - | 13 |
| Rupture, etc. - - - - - | 10 |
| Injuries received in the service - - - - | 5 |
| Ague - - - - - - | 1 |
| Diseases of the liver or kidneys, deafness, lameness, or other infirmities - - - - - | 25 |
| Abolition of their office - - - - | 6 |
| At petitioners' own request, having completed 50 years service - | 3 |
| | 225 |

**Removable Causes of Disease.** In my last annual report I stated, that the passages and hall on the secretary's side were extremely cold and draughty during the winter season, and I recommended that a fireplace or stove for warming the ascending air should be erected at the bottom of the grand staircase. This has been done, and I have reason to believe that it has had all the good effect anticipated from it. Complaints of colds and attacks of rheumatism from the officers on that side have been much less frequent this year than formerly.

With the exception of the draughts of cold air, which in the winter very much incommode officers doing sorting duties in some parts of the inland and newspaper offices, and which, from the construction of the building itself, are very difficult, if not impossible, wholly to get rid of, without interfering with the dispatch of public business, most of the removable causes of disease to which the men are exposed, are to be found in the insalubrity, the ill-ventilated, undrained, overcrowded, or confined condition of their private dwellings, with which, of course, the medical officer has no power to interfere except by way of advice.

One among many examples may be mentioned, of a letter-carrier whom I attended for disease of the lungs. I found him, with his wife and child, sleeping in a close room, or rather box, measuring six feet in length, five in width, by eight in height. This gives an amount of only 240 cubic feet of air among the three, iucluding the space occupied by the furniture, whereas there should be at least 2000 cubic feet. There was no window or fireplace to the apartment, if such it may be termed. The only mode by which it was supplied with fresh air, was a broken window adjacent to the door, and to which I attribute his very dangerous illness. It should be borne in mind, when estimating the injurious effects of living in overcrowded, or in close, unventilated rooms, that the mischief is by no means entirely owing to the absence of the oxygen and its replacement by carbonic acid gas. Animals kept in a limited space, where the normal chemical constitution of the atmosphere is kept up by the absorption of the carbonic acid gas as soon as formed, and the substitution of oxygen, nevertheless quickly die from the effects of the gaseous and other effete matters thrown off from the lungs and skins of their own bodies.

Ventilation of the Buildings. I have approved of a plan for ventilating the chief office, suggested by Mr. Cowper, C.E., which we hope will supply all the offices with a sufficient amount of fresh air, without causing draughts. If this very difficult object can be attained, I have little doubt that the health of the officers in general will be greatly promoted thereby. As the plan has received the sanction of the Lords of the Treasury, and the necessary instructions have been given to the Clerk of the Works, I hope it will be in full operation by the ensuing summer.

Provincial and Country Offices. In addition to suggestions for the improvement of different rooms and offices connected with the chief, money-order, and branch offices in

London, all of which have been attended to, and the recommendations carried out, during the course of the year I visited the following offices, some of them by special request of his Grace the Postmaster-General, and made reports as to their sanitary condition:—Edinburgh, which is about to be abandoned as soon as a new office can be erected, Glasgow, Liverpool, Manchester, Cardiff, and Bristol.

I have the honour to remain, Sir,

Your obedient servant,

WALLER LEWIS, M.B.Cantab., F.G.S., etc.

---

